# Clinical Characteristics and Outcomes of Patients Undergoing Pericardiocentesis for Pericardial Effusion: A Single-Center Experience with Exploratory Inflammatory Biomarker Analysis

**DOI:** 10.3390/jcm15197679

**Published:** 2026-10-04

**Authors:** Zeynep Yapan Emren, Gamze Erkılınç

**Affiliations:** 1 Department of Cardiology, Bakırçay University Çiğli Training and Research Hospital, Izmir 35620, Türkiye; 2 Department of Pathology, Bakırçay University Çiğli Training and Research Hospital, Izmir 35620, Türkiye

**Keywords:** pericardial effusion, pericardiocentesis, malignancy, clinical outcomes, inflammation, pan-immune–inflammation value

## Abstract

**Objective**: We aim to characterize patients undergoing pericardiocentesis for clinically significant pericardial effusion and explore differences in inflammation-based biomarkers according to malignancy status and one-year outcome. **Methods**: We retrospectively evaluated 113 consecutive patients who underwent pericardiocentesis between January 2021 and January 2026. Clinical, laboratory, pericardial fluid, cytopathological, recurrence, and mortality data were analyzed. NLR, PLR, SII, SIRI, and PIV were assessed as secondary exploratory biomarkers. **Results**: Malignancy-associated pericardial effusion was present in 36 patients (31.9%), while 77 (68.1%) had non-malignant effusion. The malignancy-associated group had more frequent dyspnea (77.8% vs. 55.8%; *p* = 0.025), effusion >15 mm (80.6% vs. 58.4%; *p* = 0.021), hemorrhagic fluid (75.0% vs. 54.5%; *p* = 0.038), and recurrence (38.9% vs. 20.8%; *p* = 0.042). Malignant cells were identified in pericardial fluid cytology in 15/36 patients (41.7%). One-year mortality was 22.2% versus 13.0% (*p* = 0.211). NLR, PLR, SII, SIRI, and PIV did not differ significantly according to malignancy status or one-year mortality; NLR, PLR, and SIRI were higher in the exploratory lung cancer subgroup. **Conclusions**: Malignancy-associated pericardial effusion showed distinct clinical and fluid characteristics. No statistically significant associations were detected between the evaluated inflammation-based biomarkers and malignancy status or one-year mortality; however, the exploratory design and limited number of outcome events warrant cautious interpretation.

## 1. Introduction

Pericardial effusion is a clinically heterogeneous disease that may be caused by a wide range of diseases like malignancy, infection, autoimmune disease, renal failure, heart failure, metabolic disorders and iatrogenic causes. The clinical significance of the fluid accumulated is determined not only by the volume but also by the rate of accumulation and the hemodynamic effects. The most serious complication is cardiac tamponade, a potentially life-threatening condition in which increased intrapericardial pressure impairs cardiac filling and hemodynamic stability. Pericardiocentesis therefore has an important therapeutic role in patients with hemodynamically significant effusions, and can also provide useful diagnostic information by biochemical, microbiological and cytopathological analysis of pericardial fluid [1,2].

Malignancy represents an important cause of clinically significant pericardial effusion and is particularly relevant in patients requiring drainage. Malignant pericardial involvement may be due to direct tumor invasion, metastatic spread, impaired lymphatic drainage or treatment-related mechanisms. Malignant pericardial effusion is common in patients with advanced disease, and early identification of clinical features associated with a malignant etiology may guide diagnostic evaluation and subsequent management [3,4,5].

Nevertheless, differentiating malignant from non-malignancy-associated pericardial effusion may be challenging. Cytopathological examination is an important component of the diagnostic work-up, but malignant cells are not invariably detected in pericardial fluid. Consequently, the overall clinical presentation, imaging findings, characteristics of the pericardial fluid, recurrence pattern, and oncological assessment should be considered together when determining etiology.

Systemic inflammatory activation represents another potential source of clinically relevant information in patients with pericardial disease. Several indices derived from routine complete blood count parameters, including NLR, PLR, SII, SIRI, and PIV, have been investigated in oncological and cardiovascular disorders [6,7,8,9,10,11]. These indices incorporate different components of the inflammatory and immune response and have been associated with disease severity and prognosis in several clinical settings [12,13,14,15,16,17,18].

PIV is one of the more recently introduced composite inflammatory indices and integrates neutrophil, monocyte, platelet, and lymphocyte counts into a single measure. Elevated PIV has been associated with adverse outcomes in several malignancies and cardiovascular diseases [13,15,16,17,18]. However, the clinical relevance of PIV and other inflammation-based indices in patients presenting with significant pericardial effusion remains insufficiently characterized.

Rather than considering systemic inflammatory markers as isolated diagnostic indicators of malignancy, their evaluation may provide complementary information within the broader clinical characterization of patients undergoing pericardiocentesis.

Accordingly, the primary aim of this study was to describe the clinical, laboratory, pericardial fluid, and outcome characteristics of patients undergoing pericardiocentesis for clinically significant pericardial effusion in a single-center cohort, with particular emphasis on differences between malignancy-associated and non-malignancy-associated effusions. As a secondary exploratory objective, we evaluated NLR, PLR, SII, SIRI, and PIV according to malignancy status, one-year mortality, and malignancy subtype.

## 2. Materials and Methods

### 2.1. Study Design and Patient Population

We conducted a retrospective single-center study including 113 consecutive patients who underwent pericardiocentesis for clinically significant pericardial effusion between January 2021 and January 2026.

### 2.2. Inclusion and Exclusion Criteria

Eligible patients were ≥18 years of age, underwent pericardiocentesis for clinically significant pericardial effusion, had available pericardial fluid cytology results, and underwent evaluation for an underlying malignant etiology. Pericardial effusion size was assessed by transthoracic echocardiography using the maximal end-diastolic echo-free space. According to the 2025 ESC classification, effusions are categorized as mild (<10 mm), moderate (10–20 mm), and large (>20 mm) [19]. The >15-mm threshold in the present study was a prespecified variable available in the retrospective dataset and was retained for comparative analyses; it was not used as a synonym for guideline-defined large pericardial effusion.

The decision to perform pericardiocentesis was based on the clinical and haemodynamic assessment documented by the treating team. Contemporary ESC guidance supports pericardiocentesis for cardiac tamponade, symptomatic moderate-to-large effusions not responding to medical therapy, and when pericardial fluid analysis is required for etiological evaluation, including suspected malignancy or bacterial infection [19].

Patients were classified as having malignancy-associated pericardial effusion when a documented diagnosis of malignancy was present at the time of pericardiocentesis, irrespective of pericardial fluid cytology. Positive pericardial fluid cytology was considered evidence of cytologically confirmed malignant pericardial involvement but was not required for classification into the malignancy-associated group.

Patients younger than 18 years, those who did not undergo pericardiocentesis, those without cytopathological examination of pericardial fluid, and patients with advanced heart failure were excluded.

### 2.3. Clinical and Laboratory Data

Hospital records were retrospectively reviewed to obtain demographic characteristics, clinical presentation, comorbidities, electrocardiographic findings, echocardiographic parameters, hematological and biochemical measurements, pericardial fluid characteristics, cytopathological findings, recurrence, and one-year mortality.

Patients were categorized according to the presence or absence of a documented malignancy at the time of pericardiocentesis. Clinical and laboratory characteristics and pericardial fluid findings were compared between the malignancy-associated and non-malignant groups.

The frequency of recurrent pericardial effusion, defined as ≥2 episodes, was also evaluated. One-year mortality was assessed as a clinical outcome.

### 2.4. Assessment of Inflammation-Based Biomarkers

As a secondary exploratory analysis, systemic inflammation was assessed using indices derived from routine complete blood count parameters obtained at presentation before pericardiocentesis.

The neutrophil-to-lymphocyte ratio (NLR) was calculated by dividing the absolute neutrophil count by the lymphocyte count.

The platelet-to-lymphocyte ratio (PLR) was calculated by dividing the platelet count by the lymphocyte count.

The systemic immune-inflammation index (SII) was calculated as: SII = platelet count × neutrophil count/lymphocyte count.

The systemic inflammation response index (SIRI) was calculated as: SIRI = neutrophil count × monocyte count/lymphocyte count.

The pan-immune–inflammation value (PIV) was calculated as: PIV = neutrophil count × platelet count × monocyte count/lymphocyte count.

For calculation of the composite indices, absolute neutrophil, lymphocyte, monocyte, and platelet counts were expressed consistently in ×10^3^/µL before application of the formulas, in accordance with the cited literature. All inflammatory indices were derived from baseline laboratory measurements obtained before pericardiocentesis.

Inflammation-based biomarkers were compared between malignancy-associated and non-malignancy-associated pericardial effusions and according to one-year mortality status in the overall cohort (*n* = 113). One-year mortality was analyzed as all-cause mortality. An additional exploratory subgroup analysis compared patients with lung cancer-associated pericardial effusion with patients with other malignancies.

### 2.5. Ethical Considerations

The study was approved by the Non-Interventional Ethics Committee of Izmir Bakırçay University (approval date: 14 May 2025; Decision No. 2257).

### 2.6. Statistical Analysis

Statistical analyses were performed using IBM SPSS Statistics for Windows (IBM Corp., Version 20, Armonk, NY, USA).

The distribution of continuous variables was assessed using the Shapiro–Wilk test and visual inspection of histograms. Normally distributed variables were expressed as mean ± standard deviation (SD), whereas non-normally distributed variables were presented as median and interquartile range (IQR). Categorical variables were reported as numbers and percentages.

Categorical variables were compared using Pearson’s chi-square test when expected cell counts were adequate and Fisher’s exact test when expected cell counts were small. For Mann–Whitney U comparisons of the principal biomarker analyses, rank–biserial correlation was reported as an effect-size estimate with bootstrap 95% confidence intervals.

Patients were categorized according to malignancy status, one-year mortality status, and cancer subtype (lung cancer versus other malignancies). Differences in inflammation-based biomarkers, including NLR, PLR, SII, SIRI, and PIV, were evaluated between these groups.

All statistical tests were two-tailed, and a *p* value < 0.05 was considered statistically significant.

Given the exploratory nature of the study, no formal adjustment for multiple comparisons was applied. Individual p values should therefore be interpreted cautiously, and these analyses should be regarded as hypothesis-generating rather than confirmatory.

## 3. Results

### 3.1. Study Population and Etiology

The study included 113 patients who underwent pericardiocentesis for clinically significant pericardial effusion. Of these, 36 patients (31.9%) had a documented malignancy at the time of pericardiocentesis and were classified as having malignancy-associated pericardial effusion, whereas 77 patients (68.1%) had non-malignant effusion. Malignant cells were identified in pericardial fluid cytology in 15 of the 36 malignancy-associated cases (41.7%); the remaining 21 patients had documented malignancy but negative pericardial fluid cytology.

Among the 77 patients with non-malignancy-associated pericardial effusion, the most common etiology was infectious disease (*n* = 31, 40.3%), followed by idiopathic (*n* = 22, 28.6%) and uremic causes (*n* = 19, 24.7%). Autoimmune disease and post-cardiac injury syndrome accounted for one case each (1.3%), while three patients (3.9%) had other non-malignant etiologies (Table 1).

Median age was comparable between the malignancy-associated and non-malignant groups (62 [IQR 51–68] vs. 66 [IQR 52–76] years; *p* = 0.139). No significant differences were observed in white blood cell, neutrophil, lymphocyte, monocyte, or platelet counts. Hemoglobin levels and left ventricular ejection fraction were also comparable between groups (Table 2).

### 3.2. Clinical and Pericardial Fluid Characteristics

Chronic kidney disease was significantly more common in the non-malignant group (23.4% vs. 2.8%; *p* = 0.006). Congestive heart failure occurred only in the non-malignant group (11.7% vs. 0%), but this difference was not statistically significant using Fisher’s exact test (*p* = 0.055).

No statistically significant between-group differences were observed for male sex or sinus rhythm on ECG (both *p* > 0.05). Anticoagulant use was less frequent in the malignancy-associated group (13.9% vs. 46.8%; *p* < 0.001).

### 3.3. Exploratory Analysis of Inflammation-Based Biomarkers

Inflammation-based biomarkers were evaluated as a secondary exploratory component of the study. No statistically significant differences in NLR, PLR, SII, SIRI, or PIV were observed between the malignancy-associated and non-malignant groups (Table 3).

Median NLR was 3.5 (IQR 2.2–5.5) in the malignancy-associated group and 3.2 (IQR 2.05–6.17) in the non-malignant group (*p* = 0.907). Similarly, no significant differences were observed for PLR (*p* = 0.936), SII (*p* = 0.779), SIRI (*p* = 0.239), or PIV (*p* = 0.289).

One-year mortality analysis was performed in the overall cohort (*n* = 113), comprising 95 patients classified as survivors and 18 as non-survivors. No statistically significant differences were detected in NLR, PLR, SII, SIRI, or PIV between survivors and non-survivors (all *p* > 0.05; Table 4). Effect-size confidence intervals were relatively wide, reflecting the limited number of deaths and indicating that clinically relevant associations cannot be excluded.

These findings indicate that, within this cohort, routinely derived systemic inflammatory indices were not significantly associated with malignancy status or one-year mortality.

### 3.4. Exploratory Analysis According to Malignancy Subtype

Among the 36 patients with malignancy-associated pericardial effusion, 17 (47.2%) had lung cancer and 19 (52.8%) had other malignancies. Detailed information on lung cancer histological subtype, disease stage, active oncological treatment, and immune checkpoint inhibitor exposure was not consistently available in the retrospective records. Inflammation-based biomarkers were compared between the lung cancer and other-malignancy subgroups (Table 5), and these comparisons were considered exploratory.

## 4. Discussion

The present study provides a single-center real-world description of patients undergoing pericardiocentesis for clinically significant pericardial effusion. Approximately one-third of the cohort had a documented malignancy at the time of pericardiocentesis. Compared with the non-malignant group, the malignancy-associated group had more frequent dyspnea, effusion >15 mm, hemorrhagic pericardial fluid, and recurrent effusion, whereas anticoagulant use was less frequent. Malignant cells were identified in pericardial fluid cytology in 41.7% of patients in the malignancy-associated group. One-year mortality did not differ significantly between groups. In secondary exploratory analyses, the evaluated inflammation-based biomarkers were not significantly associated with malignancy status or one-year mortality, while NLR, PLR, and SIRI were higher in the lung cancer subgroup than in patients with other malignancies.

Malignancy is an important clinical context in patients with pericardial effusion requiring drainage. Direct malignant pericardial involvement may occur through local invasion or metastatic dissemination, while effusion in patients with cancer may also reflect impaired lymphatic drainage, treatment-related mechanisms, infection, or other causes [3,4,5]. In the present cohort, 31.9% of patients undergoing pericardiocentesis had a documented malignancy, demonstrating the substantial contribution of cancer-associated presentations to clinically important pericardial disease.

Several readily identifiable clinical characteristics differed between the malignancy-associated and non-malignant groups. Dyspnea was significantly more common in the malignancy-associated group. This finding may reflect a greater pericardial fluid burden, as well as the contribution of the underlying cancer to respiratory symptoms. Although dyspnea is inherently nonspecific, its greater frequency may provide useful contextual information when interpreted together with imaging and pericardial fluid findings.

Effusion size also differed significantly according to malignancy status. Pericardial effusion >15 mm was observed in 80.6% of the malignancy-associated group compared with 58.4% of patients with non-malignant disease. The >15-mm threshold should be interpreted as a study-specific comparative variable rather than a guideline-defined category of large effusion. Greater fluid accumulation in an appropriate clinical context may raise suspicion of malignancy-associated disease, although effusion size alone cannot establish malignant pericardial involvement. The characteristics of the drained pericardial fluid provided additional clinically relevant information. Hemorrhagic fluid was significantly more common in the malignancy-associated group than in the non-malignant group. Hemorrhagic appearance is not specific for malignancy and should therefore be considered a supportive finding rather than a diagnostic criterion in isolation [3,4,5].

Recurrence also differed between groups, occurring in 38.9% of patients with documented malignancy and 20.8% of patients without malignancy. Persistent or recurrent effusion in a patient with cancer may have several mechanisms and warrants continued clinical assessment. Cytopathological examination remains central when malignant pericardial involvement is suspected; however, malignant cells were identified in only 41.7% of the malignancy-associated group. Thus, negative cytology did not confirm malignant involvement in the remaining patients, and the results should be interpreted together with oncological history, clinical presentation, imaging, and follow-up [5,20].

Importantly, cytology-negative pericardial disease with infiltrative or malignant-appearing features is not specific to malignancy. Rare non-neoplastic infiltrative disorders, such as Erdheim–Chester disease, may present with pericardial effusion and tumor-like cardiac or pericardial involvement, thereby mimicking malignant disease. These conditions should be considered in selected patients when cytological findings are negative or inconclusive despite persistent clinical or imaging suspicion [21].

Although one-year mortality was numerically higher in patients with malignancy-associated compared with non-malignant effusion (22.2% vs. 13.0%), this difference did not reach statistical significance. The outcome of pericardial effusion is likely determined by multiple factors like the underlying disease, type and stage of cancer, oncological treatment, hemodynamic status, comorbidities and recurrence. The number of mortality events in the present cohort may also have limited the statistical power to demonstrate differences in one-year mortality.

As a secondary exploratory component of the study, we assessed several inflammation-based biomarkers derived from routine complete blood count parameters. Systemic inflammation is increasingly recognized as an important component of both cardiovascular disease and cancer biology. Neutrophils, platelets, and monocytes participate in inflammatory signaling, tumor progression, angiogenesis, and metastatic dissemination, whereas lymphocyte depletion may reflect impaired antitumor immune surveillance [22,23]. Composite indices such as NLR, PLR, SII, SIRI, and PIV have consequently been investigated as prognostic biomarkers across a range of malignant and cardiovascular conditions [6,7,8,9,10,11,12,13,14,15,16,17,18].

In the present cohort, however, none of these indices differed significantly between the malignancy-associated and non-malignant groups. The non-malignant group was etiologically heterogeneous, with infectious, idiopathic, and uremic causes accounting for most cases. Infectious and uremic conditions may independently alter circulating inflammatory cell counts and derived indices, potentially reducing the etiological specificity of NLR, PLR, SII, SIRI, and PIV in this setting. Consequently, routine systemic inflammatory indices may have limited etiological specificity in patients requiring pericardiocentesis.

The prognostic relevance of inflammation-based indices has also been demonstrated in other cardiovascular settings. For example, Trimarchi et al. reported an association between the Advanced Lung Cancer Inflammation Index and all-cause mortality in patients with ST-elevation myocardial infarction undergoing primary percutaneous coronary intervention [24]. Although this population differs substantially from patients with pericardial effusion, these findings illustrate the broader prognostic interest in inexpensive inflammation- and nutrition-based indices.

PIV, which incorporates neutrophil, platelet, monocyte, and lymphocyte counts into a single composite index, has emerged as a prognostic marker in several malignancies since its introduction by Fucà et al. [13,15,25]. In our cohort, PIV did not differ significantly between the malignancy-associated and non-malignant groups and was not significantly associated with one-year mortality. These findings do not establish absence of diagnostic or prognostic relevance but indicate that PIV was not significantly associated with malignancy status or one-year mortality in this exploratory cohort. The malignancy subtype analysis yielded an additional observation. Patients with lung cancer-associated pericardial effusion had higher NLR, PLR, and SIRI values than patients with other malignancies, whereas SII and PIV were numerically but not significantly higher. Lung cancer is associated with systemic inflammation through several mechanisms, including smoking-related inflammation, cytokine signaling, and tumor–immune interactions [6,7]. Lung cancer is also a common malignancy among patients presenting with pericardial effusion [3,5,26]. Because the subgroup was small and detailed stage, histology, and treatment data were unavailable, these findings should be considered hypothesis-generating and require confirmation. Overall, evaluation of patients requiring pericardiocentesis should remain centered on clinical presentation, effusion characteristics, cytopathology, imaging, recurrence pattern, and underlying disease.

Several limitations should be acknowledged. First, the retrospective and single-center design may have introduced selection bias and limits the generalizability of the findings. Second, classification of the malignancy-associated group was based on a documented malignancy diagnosis rather than cytological confirmation in all patients; only 15 of 36 patients had malignant cells identified in pericardial fluid. Therefore, direct malignant pericardial involvement could not be confirmed in the 21 cytology-negative cases, and some degree of etiological misclassification cannot be excluded. Third, no a priori sample-size calculation was performed because all eligible patients during the predefined study period were included; the modest sample size and limited number of one-year deaths reduced statistical precision and precluded a robust extensively adjusted multivariable model. Fourth, inflammatory biomarkers were derived from a single baseline blood sample, and temporal changes could not be evaluated. Fifth, the non-malignant group comprised heterogeneous etiologies that may independently influence systemic inflammatory marker levels. Sixth, multiple exploratory comparisons were performed without formal adjustment for multiplicity; therefore, statistically significant subgroup findings should be regarded as hypothesis-generating. In addition, the duration and temporal evolution of pericardial effusion could not be reliably classified from the available records. Detailed lung cancer histological subtype, stage, active oncological treatment, and immune checkpoint inhibitor exposure were not consistently available, and cause-specific mortality data were incomplete. Accordingly, the absence of statistically significant biomarker associations should not be interpreted as definitive evidence of absent diagnostic or prognostic relevance, and the findings require validation in larger prospective cohorts.

## 5. Conclusions

A documented malignancy was present in approximately one-third of patients undergoing pericardiocentesis in this single-center series. The malignancy-associated group had higher rates of dyspnea, effusion >15 mm, hemorrhagic pericardial fluid, and recurrence, while anticoagulant use was less frequent. These findings may provide complementary clinical information but do not establish malignant pericardial involvement in the absence of cytological or histological confirmation. No statistically significant associations were detected between NLR, PLR, SII, SIRI, or PIV and malignancy status or one-year mortality in this cohort; however, the limited sample size and number of deaths mean that clinically relevant associations cannot be excluded. Higher NLR, PLR, and SIRI values in the lung cancer subgroup should be considered exploratory and require confirmation in larger, well-characterized prospective cohorts.

## Figures and Tables

**Table 1:** Etiological distribution of non-malignancy-associated pericardial effusion.

Non-Malignant Etiology	*n*	%
Infectious	31	40.3
Idiopathic	22	28.6
Uremic	19	24.7
Autoimmune	1	1.3
Post-cardiac injury syndrome	1	1.3
Other	3	3.9

**Table 2:** Baseline clinical, laboratory, and pericardial fluid characteristics according to malignancy status.

Variable	Malignancy-Associated (*n* = 36)	Non-Malignant (*n* = 77)	*p* Value
Age, years	62 (51–68)	66 (52–76)	0.139
Male sex, *n* (%)	27 (75.0)	43 (55.8)	0.051
White blood cell count (/µL)	7614 ± 1716	7503 ± 2033	0.777
Neutrophil count (/µL)	5110 ± 1641	5163 ± 1757	0.880
Lymphocyte count (/µL)	1533 ± 815	1519 ± 728	0.932
Monocyte count (/µL)	707 ± 394	591 ± 340	0.113
Platelet count (×10^3^/µL)	274.6 ± 74.3	271.2 ± 82.8	0.836
Hemoglobin, g/dL	12.92 ± 1.75	12.68 ± 1.85	0.505
Left ventricular ejection fraction, %	60 (60–60)	60 (58–60)	0.091
Sinus rhythm on ECG, *n* (%)	36 (100)	70 (90.9)	0.062
Hypertension, *n* (%)	16 (44.4)	35 (45.5)	0.920
Diabetes mellitus, *n* (%)	7 (19.4)	18 (23.4)	0.639
Chronic kidney disease, *n* (%)	1 (2.8)	18 (23.4)	0.006
Congestive heart failure, *n* (%)	0 (0)	9 (11.7)	0.055
Dyspnea at presentation, *n* (%)	28 (77.8)	43 (55.8)	0.025
Anticoagulant use, *n* (%)	5 (13.9)	36 (46.8)	<0.001
Hemorrhagic pericardial fluid, *n* (%)	27 (75.0)	42 (54.5)	0.038
Exudative effusion, *n* (%)	35 (97.2)	68 (88.3)	0.120
Malignant cytology, *n* (%)	15 (41.7)	0 (0)	<0.001
Recurrent effusion (≥2 episodes), *n* (%)	14 (38.9)	16 (20.8)	0.042
Pericardial effusion >15 mm, *n* (%)	29 (80.6)	45 (58.4)	0.021
One-year mortality, *n* (%)	8 (22.2)	10 (13.0)	0.211

Data are presented as mean ± standard deviation, median (interquartile range), or number (%), as appropriate.

**Table 3:** Comparison of inflammation-based biomarkers between malignancy-associated and non-malignant pericardial effusion.

Variable	Malignancy-Associated (*n* = 36)	Non-Malignant (*n* = 77)	*p* Value	Effect Size r_rb (95% CI)
NLR	3.5 (2.2–5.5)	3.2 (2.05–6.17)	0.907	0.014 (−0.223 to 0.256)
PLR	185 (132–260)	196 (125–283)	0.936	0.010 (−0.224 to 0.249)
SII	885 (618–1584)	855 (492–1632)	0.779	−0.033 (−0.279 to 0.210)
SIRI	2.47 (1.11–4.67)	1.90 (0.98–4.67)	0.239	0.138 (−0.101 to 0.371)
PIV	664 (307–1047)	499 (235–907)	0.289	0.124 (−0.113 to 0.358)

Data are presented as median (interquartile range). Effect size is reported as rank–biserial correlation (r_rb) with bootstrap 95% confidence intervals; positive values indicate higher ranks in the malignancy-associated group.

**Table 4:** Inflammation-based biomarkers according to one-year mortality status.

Variable	Survivors (*n* = 95)	Non-Survivors (*n* = 18)	*p* Value	Effect Size r_rb (95% CI)
NLR	3.3 (2.15–5.70)	4.0 (2.30–7.08)	0.397	0.127 (−0.183 to 0.432)
PLR	190 (127–257.5)	211 (141.5–350)	0.205	0.189 (−0.112 to 0.476)
SII	803 (577–1567.5)	1233.5 (761.5–1753)	0.198	0.192 (−0.091 to 0.468)
SIRI	2.00 (1.07–3.48)	2.40 (0.70–3.68)	0.745	−0.049 (−0.368 to 0.254)
PIV	542 (269.5–918)	791 (204.5–1041.3)	0.733	0.051 (−0.268 to 0.364)

Effect size is reported as rank–biserial correlation (r_rb) with bootstrap 95% confidence intervals; positive values indicate higher ranks among non-survivors.

**Table 5:** Comparison of inflammation-based biomarkers between patients with lung cancer and other malignancies.

Variable	Lung Cancer (*n* = 17)	Other Malignancies (*n* = 19)	*p* Value
NLR	5.0 (3.0–10.2)	2.5 (1.75–4.4)	0.023
PLR	243 (149–385)	165 (97.5–220)	0.028
SII	988 (698–2345)	760 (381–1117)	0.079
SIRI	3.73 (2.49–8.3)	2.0 (1.09–2.58)	0.038
PIV	825 (628–2083)	541 (301–744)	0.066

NLR, PLR, and SIRI were significantly higher among patients with lung cancer-related pericardial effusion than among patients with other malignancies (*p* = 0.023, *p* = 0.028, and *p* = 0.038, respectively). SII and PIV were also numerically higher in the lung cancer subgroup, although these differences did not reach statistical significance (*p* = 0.079 and *p* = 0.066, respectively). Given the limited subgroup sizes and multiple biomarker comparisons, these findings should be considered exploratory and hypothesis-generating.

## Data Availability

The data supporting the findings of this study are available from the corresponding author upon reasonable request. The data are not publicly available due to privacy and ethical restrictions.

## Abbreviations

NLR	Neutrophil-to-Lymphocyte Ratio
PLR	Platelet-to-Lymphocyte Ratio
SII	Systemic Immune–Inflammation Index
SIRI	Systemic Inflammation Response Index
PIV	Pan-Immune–Inflammation Value
